# Supplementary material for: Identification of Freezing-Responsive microRNAs and Their Targets in Chinese Jujube by Small RNA and Degradome Sequencing
Source: Int J Mol Sci. 2026 Sep 4;27(17):7912. doi: 10.3390/ijms27177912 (PMC13566890; doi:10.3390/ijms27177912)
Supplement: Supplementary file 1 [file ijms-27-07912-s001.zip › Supplementary Material S2/novel_miR_129.pdf]

Provisional ID	:	novel_miR_129
Score total	:	93
Score for star read(s)	:	3.9
Score for read counts	:	85.3
Score for mfe	:	2.2
Score for randfold	:	1.6
Score for cons. seed	:	
Total read count	:	179
Mature read count	:	124
Loop read count	:	0
Star read count	:	55

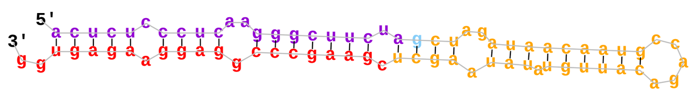

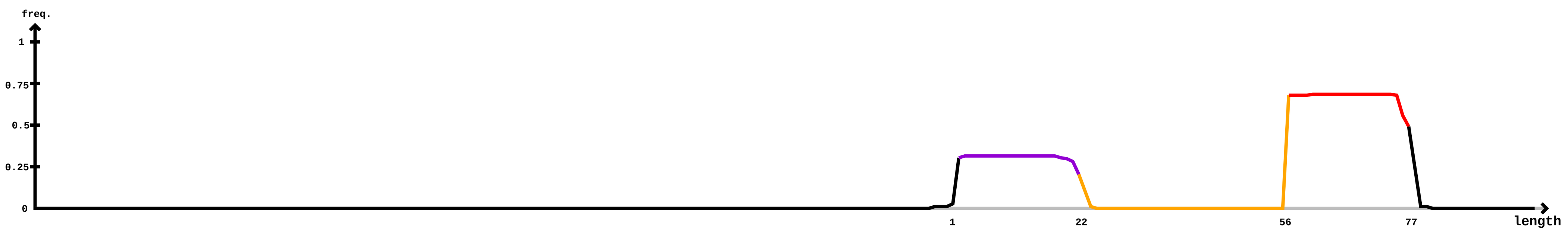

Star Mature

```
5'- agagcgaacaaaaggugucuugauauuaucauccugcucaaaggccgaucgauuaccacuaacauucguauugauuagaugaucaaguauauaaaauauauguacauguuuguauguauauauguguuggaguccaaggcauuuuuguuguucacucucccucaagggcuucuagcuagauaacaaugccagacauuguauauaagcucgaagcccggaggaagaguggacaaaggugucuugauuauau -3'   obs
    agagcgaacaaaaggugucuugauauuaucauccugcucaaaggccgaucgauuaccacuaacauucguauugauuagaugaucaaguauauaaaauauauguacauguuuguauguauauauguguuggaguccaaggcauuuuuguuguucacucucccucaagggcuucuagcuagauaacaaugccagacauuguauauaagcucgaagcccggaggaagaguggacaaaggugucuugauuauau        exp
    ...(((((.....((((...((((...(((..(((......)))..)))..)))).)))).....)))))........(((..((((.((((((..((((((((((....))))))))))))))))))))).)))((((((((((..((((((((((.((((..(((((((.((((..((((((((((.....)))))).))).)))).))))))).)))).))))))))))))))))))))......   reads   mm   sample
    .....................................................................................................................................................guucacucucccucaagggcu................................................................................   2   0   LT-1
    ........................................................................................................................................................cacucucccucaagggcuucu.............................................................................   3   0   LT-3
    .........................................................................................................................................................acucucccucaagggcuu...............................................................................   1   0   JCK-2
    .........................................................................................................................................................acucucccucaagggcuuc..............................................................................   3   0   LT-3
    .........................................................................................................................................................acucucccucaagggcuucu.............................................................................   1   0   JCK-3
    .........................................................................................................................................................acucucccucaagggcuucu.............................................................................   1   0   LT-3
    .........................................................................................................................................................Ccucucccucaagggcuucu.............................................................................   1   1   LT-2
    .........................................................................................................................................................acucucccucaaCggcuucu.............................................................................   1   1   LT-1
    .........................................................................................................................................................acucucccucaagggcuucu.............................................................................   3   0   LCK-3
    .........................................................................................................................................................acucucccucaagggcuucu.............................................................................   2   0   LT-1
    .........................................................................................................................................................acucucccucaagggcuucu.............................................................................   1   0   JCK-2
    .........................................................................................................................................................acucucccucaagggcuucu.............................................................................   1   0   LT-2
    .........................................................................................................................................................acucucccucaagggcuucua............................................................................   1   0   JCK-3
    .........................................................................................................................................................acucucccucaagggcuucua............................................................................   5   0   LT-3
    .........................................................................................................................................................acucucccucaaCggcuucua............................................................................   1   1   LT-1
    .........................................................................................................................................................acucucccucaagggcuucua............................................................................   13   0   LCK-3
    .........................................................................................................................................................acucucccucaaCggcuucua............................................................................   1   1   JCK-3
    .........................................................................................................................................................acucucccucaagggcuucua............................................................................   1   0   JCK-1
    .........................................................................................................................................................acucucccucaagggcuucua............................................................................   3   0   LT-1
    .........................................................................................................................................................acucucccucaagggcuucuag...........................................................................   2   0   LT-3
    .........................................................................................................................................................acucucccucaaCggcuucuag...........................................................................   2   1   LT-1
    .........................................................................................................................................................acucucccucaagggcuucuag...........................................................................   2   0   JCK-3
    .........................................................................................................................................................acucucccucaagggcuucuag...........................................................................   4   0   JCK-2
    ..........................................................................................................................................................cucucccucaagggcuucuagc..........................................................................   1   0   LT-1
    ..........................................................................................................................................................cucucccucaagggcuucuagc..........................................................................   1   0   JCK-3
    ................................................................................................................................................................................................................cgaagcccggaggaagag........................   1   0   LT-3
    ................................................................................................................................................................................................................cgaagcccggaggaagagu.......................   12   0   LCK-3
    ................................................................................................................................................................................................................cgaagcccggaggaagagu.......................   2   0   LT-3
    ................................................................................................................................................................................................................cgaagcccggaggaagagu.......................   8   0   LT-2
    ................................................................................................................................................................................................................cgaagcccggaggaagagug......................   6   0   LCK-3
    ................................................................................................................................................................................................................cgaagcccggaggaagagug......................   4   0   LT-2
    ................................................................................................................................................................................................................cgaagcccggaggaagagug......................   1   0   LT-3
    ................................................................................................................................................................................................................cgaagcccggaggaagagug......................   1   0   LT-1
```

```
                                                                                                                                                                Star                                                     Mature
agagcgaacaaaaggugucuugauauuaucauccugcucaaaggccgaucgauuaccacuaacauucguauugauuagaugaucaaguauauaaauauauguacauguuuguauguauauauguguuggaguccaaggcauuuuuguuguucacucucccucaagggcuucuagcuagauaacaaugccagacauuguauauaagcucgaagcccggaggaagaguggacaaaaggugucuugauuauau
...............................................................................................................................................................................................................cgaagcccggaggaagagugg......................	9	0	JCK-2
...............................................................................................................................................................................................................cgaagcccggaggaagagugg......................	34	0	LT-3
...............................................................................................................................................................................................................cgaagcccggaggaagagugg......................	4	0	JCK-1
...............................................................................................................................................................................................................cgaagcccggaggaagagugg......................	17	0	LT-1
...............................................................................................................................................................................................................cgaagcccggaggaagagugg......................	13	0	LT-2
...............................................................................................................................................................................................................cgaagcccggaggaagagugg......................	10	0	JCK-3
...............................................................................................................................................................................................................cgaagcccggaggaagaguggaAa...................	1	1	LT-2
...................................................................................................................................................................................................................gcccggaggaagaguggaca...................	1	0	LT-2
```
